# Characterization of Genomic Diversity among Carbapenem-Resistant *Escherichia coli* Clinical Isolates and Antibacterial Efficacy of Silver Nanoparticles from Pakistan

**DOI:** 10.3390/microorganisms10112283

**Published:** 2022-11-17

**Authors:** Aamir Jamal Gondal, Nakhshab Choudhry, Hina Bukhari, Zainab Rizvi, Nighat Yasmin

**Affiliations:** 1 Department of Biomedical Sciences, King Edward Medical University, Lahore 54000, Pakistan; 2 Department of Biochemistry, King Edward Medical University, Lahore 54000, Pakistan; 3 Department of Pathology, King Edward Medical University, Lahore 54000, Pakistan; 4 Department of Oral Pathology, de’Montmorency College of Dentistry, Lahore 54000, Pakistan

**Keywords:** *Escherichia coli* (*E. coli*), ST405, ST131, carbapenemases, silver nanoparticles

## Abstract

The emergence of carbapenem-resistant *Escherichia coli* (*E. coli*) is considered an important threat to public health resulting in resistance accumulation due to antibiotics misuse and selection pressure. This warrants periodic efforts to investigate and develop strategies for infection control. A total of 184 carbapenem-resistant clinical strains of *E. coli* were characterized for resistance pattern, resistance genes, plasmids, sequence types and in vitro efficacy of silver nanoparticles (AgNPs). Carbapenem resistance was prevalent in *E. coli* isolated from female patients (64.7%), urine samples (40.8%) and surgical wards (32.1%). Polymyxin-B showed higher susceptibility. ESBLs and carbapenemases were produced in 179 and 119 isolates, respectively. Carbapenemase-encoding genes were observed among 104 strains with *bla*_NDM-1_ (45.1%), *bla*_OXA-48_ (27%), *bla*_NDM-7_ (3.8%), *bla*_NDM-1_/*bla*_OXA-48_ (15.4%), *bla*_NDM-7_/*bla*_OXA-48_ (2.9%), *bla*_OXA-48_/*bla*_VIM_ (3.8%) and *bla*_NDM-1_/*bla*_VIM_ (2%). ESBL resistance genes were detected in 147 isolates, namely *bla*_SHV_ (24.9%), *bla*_CTX-M_ (17.7%), *bla*_TEM_ (4.8%), *bla*_SHV_/*bla*_CTX-M_ (29.2%), *bla*_SHV_/*bla*_TEM_ (15%) and *bla*_CTX-M_/*bla*_TEM_ (8.8%). ST405 (44.4%) and ST131 (29.2%) were more frequent sequence types with ST101 (9.7%), ST10 (9.7%) and ST648 (7%). The replicon types IncFII, IncFIIK, IncA/C, IncN and IncL/M were detected. The combination of MEM/AgNPs remained effective against carbapenemase-positive *E. coli*. We reported genetically diverse *E. coli* strains coharboring carbapenemases/ESBLs from Pakistan. Moreover, this study highlights the enhanced antibacterial activity of MEM/AgNPs and may be used to manage bacterial infections.

## 1. Introduction

The repeated exposure of bacterial species to antibiotics results in selection pressure ultimately modifying their antimicrobial physiology. Carbapenem resistance has been accumulated among the Enterobacteriaceae (CRE) due to the significant spread of core carbapenemase-encoding genes present on plasmids or mobile genetic elements such as integrons and transposons. Therefore, carbapenemases presented a stable and transferable form of resistance due to carbapenem hydrolysis and geographic spread such as *bla*_NDM_, *bla*_OXA-48_, *bla*_VIM_, *bla*_IMP_ and *bla*_KPC_ [1,2]. Since the identification of *bla*_NDM_, its spread has been commonly observed among Enterobacteriaceae, especially from Asian territories such as Pakistan, India and China. *bla*_IMP_-carrying CRE were predominantly detected in Taiwan and Japan with worldwide sporadic reports. Greece was identified as a center of *bla*_VIM_-positive CRE leading to the outbreaks in Europe and China. On the other hand, the eruption of *bla*_KPC_-producing CRE was recorded in the USA, Europe and China, while Turkey, Europe and the Mediterranean region are the core places for OXA-48-producing CRE [3,4]. This geographical distribution of carbapenemases showed that CRE members have mastered the art of hiding through interchangeable resistance features responsible for the dispersion of carbapenem resistance. Common CRE pathogens responsible for amplified resistance spread through the distribution of carbapenemases, including *E. coli*, *K. pneumoniae* and *Enterobacter* spp. [1,2]. Therefore, the detection and pursuing of such pathogens has been recommended as a critical priority by the CDC and WHO [4].

*E. coli* is a part of normal human gut microbiota; however, several supremely adapted *E. coli* clones with multiple resistant genes were shown to accustom the new niches, thus causing far-ranging diseases such as urinary/respiratory tract infections, intestinal infections and sepsis [5,6]. Most studies reported antimicrobial resistance among *E. coli* in the non-clinical settings from Pakistan such as *bla*_OXA-1_, *bla*_CTX-M15_ and *bla*_TEM_ in chicken meat [7], ST10 with mcr-1 and *bla*_TEM_ in cattle farm environment [8] and *bla*_NDM_, *bla*_OXA-48_, *bla*_TEM_ and *bla*_SHV_ from sewage water [9]. However, there are few reports regarding high-risk clones of *E. coli* with multiple resistance genes in hospital-acquired infections from Pakistan, such as ST131, which has been linked to *bla*_KPC-2_, while ST1196 has been linked to *bla*_NDM-1_ [10].

The efficacy of the currently available antibiotics has reduced due to the emergence of resistant bacterial clones, forcing the search for other methods to combat such dangerous clones. In this regard, nanoparticles are attractive candidates due to their stability and biocompatibility, as evidenced by widespread antimicrobial, industrial and biomedical applications [11]. It was shown that nanoparticles, in combination with antibiotics, enhanced the antimicrobial efficiency against resistant microorganisms [12]. A powerful antibacterial response has been observed when myco-synthesized AgNPs in combination with imipenem were used against imipenem-resistant *K. pneumoniae* isolates. The MIC values of imipenem were reduced for imipenem-resistant *K. pneumoniae* strains with an FIC index of 0.07 [13]. Biologically synthesized AgNPs from plant extracts have been used with tetracycline against *S. aureus* and *K. pneumoniae,* showing significantly increased activity [14]. Another study reported the combined elevated effect of AgNPs with kanamycin [15] and colistin against MDR pathogens [16]. Therefore, the use of AgNPs may be considered as one of the useful therapeutic strategies for treating microbial infections and the reversal of bacterial resistance.

Globally widespread carbapenem-resistant *E. coli* strains necessitate novel approaches to stop the spread of these dangerous infections. Therefore, continuous surveillance studies and improved antimicrobial usage plans are required so that the proper measures can be adopted to overcome the spread of high-risk clones. The distribution of carbapenemases among Pakistan *E. coli* is, however, only partially and sparsely studied [17]. Therefore, constant surveillance investigations are required in terms of genotyping, plasmid and sequence typing to address the resistance situation in this country. Keeping this in view, this study aims to investigate the burden of carbapenem resistance, genomic diversity and efficacy of antimicrobials with silver nanoparticles among *E. coli* clinical isolates from Pakistan, so that applicable strategies can be devised for appropriate infection control.

## 2. Materials and Methods

A total of 184 carbapenem-resistant clinical strains of *E. coli* were collected during May-2019 till August-2020 from routine diagnostic facility of Mayo hospital, Lahore, Pakistan. Clinical samples were collected from different hospital sections such as surgery (59/184, 32.1%), nephrology (36/184, 19.6%), medicine (27/184, 14.7%), urology (23/184, 12.5%), ICU (9/184, 4.9%), pediatric medicine (9/184, 4.9%), chest medicine (8/184, 4.3%), cardiology (7/184, 3.8%) and oncology (6/184, 3.3%). Sample types included urine (75/184, 40.8%), wounds (28/184, 15.2%), pus (27/184, 14.7%), pleural fluids (17/184, 9.2%), tip cells (17/192, 9.2%), blood (13/184, 7.1%), sputum (4/184, 2.2%) and tissue (3/184, 1.6%). The clinical samples were cultured on MacConkey agar (Oxoid Ltd., Basingstoke, UK) while cysteine lactose electrolyte-deficient (CLED) media (Oxoid Ltd., Basingstoke, UK) were used for urine samples. CHROMagar^TM^ ESBL media (CHROMagar, Paris, France) were used to identify ESBL producer strains. The bacterial cultures were characterized by Gram’s staining and API-20E (BioMerieux, Marcy-l’Étoile, France).

### 2.1. Determination of Antibiotic Susceptibility Pattern

Antibiotic susceptibility testing was conducted by using the standard disc diffusion method as per CLSI instructions [18]. The antimicrobial discs meropenem (MEM; 10 µg), ertapenem (ETP; 10 µg), imipenem (IMP; 10 µg), cefzolin (CFZ; 30 µg), ceftaroline (CPT; 30 µg), cefotaxime (CTX; 30 µg), ceftazidime (CAZ; 30 µg), amikacin (AK; 10 µg), ciprofloxacin (CIP; 5 µg), ampicillin (AMP; 10 µg), doxycycline (DO; 30 µg), aztreonam (ATM; 30 µg), amoxicillin–clavulanic acid (AMC; 20/10 µg), piperacillin–tazobactam (TZP; 100/10 µg), tigecycline (TGC; 15 µg), polymyxin-B (PB; 300U) and trimethoprim–sulfamethoxazole (SXT; 1.25/23.75 µg) (Oxoid, Basingstoke, UK) were used. The standard broth microdilution method was used to ascertain antimicrobials MIC. The double-disc synergy test and carbapenem inactivation method were used to identify extended spectrum β-lactamases (ESBLs) and carbapenemase-producing strains. Quality control strains were *E. coli* ATCC 25922 and *P. aeruginosa* ATCC 27853.

### 2.2. Detection of Antimicrobial Resistance-Encoding Genes

Genomic DNA was isolated by the heat lysis method [19]. Briefly, 2 to 3 colonies of bacterial culture were mixed with 500 µL sterile distilled water and heated at 98 °C for 10 min at 300 rpm (ThermoMixer, Fischer Scientific, Waltham, MA, USA). Tubes were centrifuged at 1000 rpm for 10 min and supernatant collected in newly labeled tube. DNA was stored at −80 °C. The carbapenemase-encoding genes *bla*_NDM-1_, *bla*_OXA-48_, *bla*_IMP_, *bla*_VIM_ and *bla*_KPC-2_ and the ESBLs *bla*_CTX-M_, bla_SHV_ and *bla*_TEM_ were detected by using a 50 µL of PCR reaction mixture containing 25 µL of 2 × PCR Master Mix (Cat # K0171, Thermoscientific, Waltham, MA, USA), 1 µL of each primer (10 µM), 2 µL of DNA and dH_2_O up to 50 µL in PCR (Thermal Cycler, Proflex, ABI). PCR cycling conditions were 95 °C for 40 sec, melting temperature (Tm) 30 sec and 72 °C for 30 sec (30 cycles). Agarose gel electrophoresis (1–1.5%) was used to resolve PCR products. Allelic discrimination of *bla*_NDM_ was performed by Sanger’s sequencing method. The cycle sequencing was performed by using BigDye terminator v3.1 kit with 10 µL PCR reaction mixture containing BigDye terminator 3.1 Ready Reaction Mix 4 µL, forward primer (3.2 pmol) 0.5 µL, purified DNA template (5–20 ng) 2 µL and dH_2_O 3.5 µL. PCR cycling conditions were 96 °C for 1 min, 96 °C for 10 sec, 50 °C for 5 sec and 60 °C for 2 min (35 cycles). PCR product was purified by using BigDye XTerminator purification kit as per kit instructions and capillary electrophoresis was conducted by Genetic Analyzer (ABI-3500, Thermo Fischer, Waltham, MA, USA). Sequencing analysis software v6.1 and basic local alignment tool (BLAST, NCBI) was used for data analysis and interpretation. The primer sequences with Tm are given in Table S1.

### 2.3. Multilocus Sequence Typing (MLST) and Plasmid Analysis

MLST was used for sequence typing analysis of *E. coli* strains harboring *bla*_NDM_ based on the allelic profile resemblance of eight housekeeping genes (*E. coli* Pasteur Scheme): DNA polymerase (*dinB*), isocitrate dehydrogenase (*icdA*), p-aminobenzoate synthase (*pabB*), polymerase PolII (*polB*), proline permease (*putP*), tryptophan synthase subunit A (*trpA*), tryptophan synthase subunit B (*trpB*) and beta-glucuronidase (*uidA*) [20]. The primer sequences are given in Table S1. The same primers were used for sequencing analysis as described above. Allele number and STs were assigned by online MLST database https://pubmlst.org/bigsdb?db=pubmlst_mlst_seqdef (accessed on 16 December 2021). Plasmid analysis was carried out by using PCR-based replicon typing kit based on their incompatibility groups [21].

### 2.4. Determination of Efficacy of Silver Nanoparticles (AgNPs)

The activity of AgNPs and MEM was evaluated by the broth microdilution checkerboard method. Commercially available AgNPs (particle size: 10 nm, solution concentration 20 μg/mL in aqueous buffer containing sodium citrate as stabilizer) were used (Cat # 730785, Sigma-Aldrich, St. Louis, MO, USA). The dilutions of MEM and AgNPs were prepared in Mueller Hinton broth with final concentration ranges of 1024, 512, 256, 128, 64, 32, 16 and 8 μg/mL for MEM and 10, 5, 2.5, 1.25, 0.625 and 0.312 μg/mL for AgNPs (Table S2). Bacterial cultures were prepared at a concentration of 0.5 McFarland (equivalent to 10^8^ CFU/mL) and diluted to 1:100 (10^6^ CFU/mL). In sterile 96-well microtiter plate, each well was inoculated with 100 μL of diluted bacterial suspension and mixed with equal volumes of antibiotic solution. All tests were conducted in duplicate with a growth control without addition of antibiotics and with sodium citrate addition. The inoculated microtiter plate was incubated at 37 °C for 18 h. After incubation, the fractional inhibitory concentration index (ΣFIC) was calculated by dividing individual MIC of treatments with MIC of the combination drugs. ΣFIC value ≤1 was considered to have a synergistic, 1.1 to 2.0 indifferent and ≥2 antagonistic effect [22].

## 3. Results

### 3.1. Characteristics of Sample

In the current study, the isolates were screened and included on the basis of their carbapenem susceptibility profile. All the isolates were resistant to at least one of the carbapenems (MEM, IMP and ETP). On the basis of this selection criterion, 184 carbapenem-resistant *E. coli* strains were retrieved out of total 650 collected samples. Carbapenem-resistant samples were mainly obtained from females (119/184, 64.7%), the major sample type was urine (75/184, 40.8%) and the main hospital section was surgery (59/184, 32.1%), followed by nephrology (36/184, 19.6%). Carbapenemase production was recorded in 64.7% (119/184) of the carbapenem-resistant *E. coli* isolates, while 35.3% (65/184) did not produce carbapenemase, indicating that other mechanisms are involved for resistance development against carbapenems in these isolates. On other hand, ESBL production was observed in 97.3% (179/184) of the isolates.

### 3.2. Resistance Gene Profile

The carbapenemase-producing strains were further evaluated for the presence of resistance genes. It was observed that 87.4% (104/119) of isolates carried resistance genes with the higher prevalence of *bla*_NDM-1_ (45.1%, 47/104), while *bla*_NDM-7_ was detected in 3.8% (4/104) of strains. *bla*_OXA-48_ represented the second highest carbapenemase-resistance gene (27%, 28/104). On the other hand, the co-production of *bla*_NDM-1_ and *bla*_OXA-48_ was observed in 15.4% (16/104) of the strains. We also detected the presence of *bla*_NDM-7_/*bla*_OXA-48_ (2.9%, 3/104), *bla*_OXA-48_/*bla*_VIM_ (3.8%, 4/104) and *bla*_NDM-1_/*bla*_VIM_ (2%, 2/104). *bla*_KPC-*2*_ and *bla_IMP_* were not detected.

Furthermore, the frequency of the selected β-lactamase-encoding genes was assessed. ESBL-resistance genes were detected in 82.1% (147/184) of the isolates. The dominance was observed for *bla*_SHV_ (24.9%, 36/147), followed by *bla*_CTX-M_ (17.7%, 26/147) and *bla*_TEM_ (4.8%, 7/147), while in case of coharbored ESBL genes, *bla*_SHV_/*bla*_CTX-M_ (29.2%, 43/147) were in abundance together with *bla*_SHV_/*bla*_TEM_ (15%, 22/147) and *bla*_CTX-M_/*bla*_TEM_ (8.8%, 13/147). The distribution of resistance genes with respect to the samples and wards is given in Table 1.

### 3.3. Genetic Profiling and Antimicrobial Susceptibility Analysis

In order to identify the genetic variability based on clonal lineage among *E. coli* isolates, NDM-positive strains (*n* = 72) were subjected to a sequence type analysis. ST405 (44.4%, 32/72) and ST131 (29.2%, 21/72) were found to be the dominant sequence types. Other sequence types were also detected, including ST101 (9.7%, 7/72), ST10 (9.7%, 7/72) and ST648 (7%, 5/72). The detected replicon types included IncFII, IncFIIK, IncA/C, IncN and IncL/M.

Carbapenem-resistant clinical strains displayed a varied antimicrobial resistance profile with 100% resistance to third-generation cephalosporins, including CPT, CFZ, CAZ, CTX, FOX, ATM, AMP and AMC. The discrete susceptibility pattern for other antimicrobials exhibited a multifarious resistance profile such as TZP (76.3%), SXT (62.5%), TGC (54.1%) and CIP (51.4%), whereas the highest susceptibility was recorded for PB (16.6%) accompanied by DO (47.2%) and AK (45.8%). In order to investigate the antimicrobial resistance pattern in relation to sequence types, we classified the strains into diverse sets of isolates arbitrarily based on their resistance pattern. All isolates represented an MDR phenotype. The detailed results are given in Table 2.

Furthermore, the correlation of carbapenemase-resistance genes with sequence types was analyzed, by which we identified different combinations of resistance genes within different detected sequence types. Most importantly, we found that ST405 and ST131 were more prevalent comprising isolates with *bla*_NDM-7_/*bla*_OXA-48_ and *bla*_NDM-1_/*bla*_OXA-48_ which coharbored different combinations of ESBLs, while ST10, ST101 and ST648 were prevalent among the *bla*_NDM-1_ harboring isolates. The distribution of sequence types in relation to resistance genes is given in Table 3.

Carbapenem-resistant strains of *E. coli* containing carbapenemases (*n* = 16) were selected randomly for the determination of the AgNPs’ efficacy. Carbapenem-sensitive strains of *E. coli* (*n* = 6) were used as controls. The MIC values of bacterial cultures grown in the presence of MEM, AgNPs and MEM/AgNPs were determined. The MIC values of the cultures were the highest in the presence of MEM and AgNPs alone. However, when a combination of MEM/AgNPs was used, there was a reduction in the MIC values. The detailed results are described in Table 4.

## 4. Discussion

The eventual outcome of carbapenem resistance among Enterobacteriaceae was evidenced globally by the rapid circulation of plasmid-encoded carbapenemases, making them critical for nosocomial outbreaks. Although carbapenemase detection is essential for infection control purposes, their precise characterization among different species is helpful in clinical practice as it impacts therapeutic decisions. The presence of different clinical strains in the genetic context among *E. coli* has not been revealed in the study area. In the present study, carbapenemase production was observed in 64.7% of the *E. coli* isolates. Previously, a variable range of carbapenemase production among *E. coli* strains has been documented from Pakistan, such as 37.1% in 2015, 93% in 2018, 81% in 2019 and 22.02% in 2021 [5,10,23,24] with the observation of similar trends globally, such as 89% from China, 9.82% from Morocco and 20.2% from Germany [20,21,22]. Similarly, reports from Pakistan showed higher rates of carbapenemase production among other Enterobacteriales such as *K. pneumoniae* 27.2% (34/125) [10], 61.8% (68/110) [25], 77.7% (91/117) [26] and 82.8% [27]. On the other hand, we found 28.3% of carbapenem-resistant *E. coli* clinical isolates. Previous studies from Pakistan demonstrated an increasing trend of carbapenem resistance in *E. coli* with 6% to 37.9% resistance from 2014 until 2018, and thus identified *E. coli* as a major contributor to the carbapenem resistance in Pakistan [23,28,29]. The leading cause for the growing trend of increased carbapenem resistance is the excessive use of carbapenems that result in the survival of complex clones with highly endured resistant strains [30].

Our results indicate a high resistance to third-generation cephalosporins, while PB displayed a higher susceptibility. In agreement with our results, previous studies from Pakistan also reported higher susceptible rates for colistin and fosfomycin [10,31,32]. On the other hand, avian-derived *E. coli* isolates from Pakistan showed resistance to SXT, TE, CTX and CAZ, while chicken-originated isolates were more susceptible to chloramphenicol and lower levels of resistance against third-generation cephalosporins [33,34]. Moreover, we found that surgery (32.1%) and nephrology (19.6%) were the main hospital sections responsible for the spread of *E. coli* infections. However, the ICU has been linked to the dissemination of carbapenem-resistant strains in China [6,35].

We observed that the most widely circulating carbapenem-resistance genes were *bla*_NDM-1_ (45.1%), *bla*_NDM-7_ (3.8%) and *bla*_OXA-48_ (27%), while the co-existence of *bla*_NDM-1_/*bla*_OXA-48_ (15.4%), *bla*_NDM-7_/*bla*_OXA-48_ (2.9%) and *bla*_OXA-48_/*bla*_VIM_ (3.8%) was recorded. Data from Pakistan depicted that the main drivers of carbapenem resistance in *E. coli* are NDM and OXA-48, as evidenced by a number of reports such as *bla*_NDM-1_, *bla*_NDM-4_, *bla*_NDM-5_, *bla*_NDM-7,_ *bla*_OXA-48_ and *bla*_NDM-1_/*bla*_OXA-48_ [10,36,37,38]. Few reports described the presence of *bla*_VIM_ and *bla*_IMP_, while only one study reported the presence of *bla*_KPC-2_ with the coexistence of ESBLE genes in *E. coli* from Pakistan [5,10,24,39]. However, we reported the co-existence of *bla*_NDM-7_/*bla*_OXA-48_ and *bla*_OXA-48_/*bla*_VIM_ for the first time in *E. coli* isolates from Pakistan.

In the current analysis of *E. coli* strains coharboring carbapenemase genes, the co-existence of ESBLs was detected such as *bla*_NDM-1_/*bla*_OXA-48_/*bla*_SHV_, *bla*_NDM-1_/*bla*_OXA-48_/*bla*_CTX-M_/*bla*_TEM_, *bla*_NDM-1_/*bla*_OXA-48_/*bla*_CTX-M_/*bla*_SHV_ and *bla*_NDM-7_/*bla*_OXA-48_/*bla*_CTX-M_/*bla*_SHV_/*bla*_TEM_. Hitherto, the presence of carbapenemases and beta lactamases has been described from Pakistan including *bla*_NDM-1_/*bla*_CTXM-15_/*bla*_OXA-1_ and *bla*_NDM-1_/*bla*_CTXM-15_ [10]. In our strains, the coexistence of *bla*_NDM-7_/*bla*_OXA-48_/*bla*_CTX-M_/*bla*_SHV_/*bla*_TEM_ is described for the first time.

Antimicrobial pressure has the ability to single-out clonal lineages and plasmids with resistance determinants, resulting in an enhanced transmission capacity. In our study, the clonal lineage analysis showed the sequence types ST405 and ST131 predominantly coharboring *bla*_NDM-7_/*bla*_OXA-48_ and *bla*_NDM-1_/*bla*_OXA-48_, while ST101, ST10 and ST648 were prevalent among *bla*_NDM-1_ harboring isolates. Previously, ST131 and ST405 in *bla*_NDM-1_ and *bla*_KPC-2_ positive *E. coli* strains with IncH12 and IncN replicon types have been reported, while ST648 has been described in *bla*_NDM-7_ containing *E. coli* isolates. [10,36,40]. ST101 and ST648 were reported in NDM-positive *E. coli* isolates [41]. On the other hand, ST10 was reported in avian-derived *E. coli* isolates [33] and ST131 among poultry birds from Pakistan [42], while we observed ST10 among clinical isolates for the first time. Furthermore, we detected IncFII, IncFIIK, IncA/C, IncN and IncL/M replicon types among our study isolates. Other replicon types reported from Pakistan include IncL/M, IncA/c, Inc and IncF-II [26,33,36,43,44].

Regardless of the significant efforts for the improvement and control of infections, carbapenem-resistant bacteria remain an alarming threat. Thus, few treatment options are left due to limited resources. Mostly, β-lactam antimicrobials showed inconsequential treatment effects against carbapenem-resistant microbes [45]. Emerging clinical evidence suggests that treatment with combination therapy may be beneficial against carbapenem-resistant pathogens [46]. Since ancient times, silver is known for its antimicrobial effects; therefore, in order to overcome the resistance development by the extensive use of antibiotics, silver nanoparticles can be used as an alternative approach to antibiotic combination therapy against MDR organisms [15,47,48]. Our data indicated that the combination of MEM/AgNPs resulted in the reduction of MIC values as compared to the presence of MEM and AgNPs alone against NDM-positive *E. coli* isolates. It has been shown that AgNPs and ciprofloxacin have better antimicrobial efficiency against *E. coli* [49]. Moreover, it has been suggested that biosynthesized AgNPs may work as antimicrobials to control *E. coli* infections [50,51,52,53]. However, we reported for the first time the effect of AgNPs in combination with MEM against *E. coli* clinical isolates.

## 5. Conclusions

We reported the co-existence of *bla*_NDM-7_/*bla*_OXA-48_ and *bla*_OXA-48_/*bla*_VIM_ in *E. coli* isolates from Pakistan with a novel ST405 *E. coli* strain coharboring *bla*_NDM-7_/*bla*_OXA-48_/*bla*_CTX-M_/*bla*_SHV_/*bla*_TEM_. Moreover, ST10 was identified in clinical isolates coharboring *bla*_NDM-1_/*bla*_CTX-M_/*bla*_SHV_/*bla*_TEM_ for the first time. The resistance pattern observed in our study suggested that surprisingly powerful strains evolved in Pakistan with time, which may indicate a complicated survival mechanism, particularly in the scenario wherein antibiotics misuse is rising. However, due to funding issues, we could not explore other resistance mechanisms and invasive genes. Moreover, a large number of strains may be tested further for AgNPs synergism by time kill assay. Our results also show that the antimicrobial efficacy can be improved when used in combination with silver nanoparticles.

## Figures and Tables

**Table 1:** Molecular profile of carbapenem-resistant *E. coli* isolates.

Sample	Ward	Strain	Antimicrobial Resistance Gene
Urine	Urology/Nephrology	EC-75, EC-73, EC-95, EC-101	*bla*_NDM-1_/*bla*_OXA-48_/*bla*_SHV_/*bla*_CTX-M_
		EC-89	*bla*_NDM-7_/*bla*_OXA-48_/*bla*_SHV_/*bla*_CTX-M_
		EC-02	*bla*_NDM-1_/*bla*_OXA-48_/*bla*_SHV_
		EC-71	*bla*_NDM-1_/*bla*_VIM_/*bla*_SHV_
		EC-11, EC-67, EC-68, EC-94, EC-102	*bla*_NDM-1_/*bla*_SHV_/*bla*_CTX-M_
		EC-88	*bla*_NDM-1_/*bla*_CTX-M_/*bla*_TEM_
		EC-50	*bla*_NDM-7_/*bla*_SHV_/*bla*_CTX-M_
		EC-49, EC-63	*bla*_NDM-1_/*bla*_CTX-M_
		EC-86	*bla*_NDM-7_/*bla*_CTX-M_
		EC-48	*bla*_OXA-48_/*bla*_VIM_/*bla*_SHV_
		EC-01, EC-92	*bla*_OXA-48_/*bla*_SHV_/*bla*_CTX-M_
		EC-51, EC-79, EC-91	*bla*_OXA-48_/*bla*_SHV_
		EC-69	*bla*_OXA-48_/*bla*_TEM_
	Surgery	EC-97	*bla*_NDM-1_/*bla*_OXA-48_/*bla*_CTX-M_/*bla*_TEM_
		EC-10, EC-29	*bla*_NDM-1_/*bla*_SHV_/*bla*_CTX-M_/*bla*_TEM_
		EC-80	*bla*_NDM-1_/*bla*_CTX-M_
		EC-78	*bla*_NDM-1_/*bla*_SHV_
		EC-60	*bla*_OXA-48_/*bla*_SHV_/*bla*_CTX-M_
		EC-08, EC-82	*bla*_OXA-48_/*bla*_CTX-M_
		EC-99	*bla*_OXA-48_/*bla*_SHV_
	Medicine	EC-56	*bla*_NDM-1_/*bla*_OXA-48_/*bla*_CTX-M_/*bla*_TEM_
		EC-57	*bla*_NDM-1_/*bla*_OXA-48_/*bla*_SHV_/*bla*_CTX-M_
		EC-98	*bla*_NDM-7_/*bla*_OXA-48_/*bla*_SHV_/*bla*_CTX-M_
		EC-44, EC-77, EC-104	*bla*_NDM-1_/*bla*_SHV_/*bla*_CTX-M_
		EC-22	*bla*_NDM-1_/*bla*_CTX-M_
		EC-45	*bla*_NDM-1_/*bla*_SHV_
		EC-81	*bla*_OXA-48_/*bla*_SHV_/*bla*_CTX-M_
		EC-58	*bla*_OXA-48_/*bla*_SHV_
	Chest medicine	EC-103	*bla*_NDM-1_/*bla*_VIM_/*bla*_SHV_
Pus	Surgery	EC-17	*bla*_NDM-7_/*bla*_OXA-48_/*bla*_SHV_/*bla*_CTX-M_/*bla*_TEM_
		EC-39	*bla*_NDM-1_/*bla*_OXA-48_/*bla*_SHV_/*bla*_CTX-M_
		EC-23	*bla*_NDM-1_/*bla*_OXA-48_/*bla*_CTX-M_/*bla*_TEM_
		EC-09, EC-13, EC-31, EC-34, EC-40	*bla*_NDM-1_/*bla*_SHV_/*bla*_CTX-M_
		EC-14, EC-16	*bla*_NDM-1_/*bla*_CTX-M_
		EC-43	*bla*_NDM-1_/*bla*_SHV_
	Cardiology	EC-38	*bla*_NDM-1_/*bla*_SHV_/*bla*_CTX-M_
		EC-37	*bla*_OXA-48_/*bla*_SHV_/*bla*_CTX-M_
		EC-26	*bla*_OXA-48_/*bla*_SHV_
	Medicine	EC-27	*bla*_NDM-1_/*bla*_SHV_
Wound	Surgery	EC-33	*bla*_NDM-1_/*bla*_OXA-48_/*bla*_SHV_
		EC-12, EC-83	*bla*_NDM-1_/*bla*_CTX-M_/*bla*_TEM_
		EC-06	*bla*_OXA-48_/*bla*_VIM_/*bla*_CTX-M_
		EC-04	*bla*_OXA-48_/*bla*_SHV_/*bla*_CTX-M_
		EC-21	*bla*_NDM-1_/*bla*_CTX-M_
		EC-30	*bla*_NDM-1_/*bla*_SHV_
	Medicine	EC-15	*bla*_NDM-1_/*bla*_SHV_/*bla*_CTX-M_
		EC-07	*bla*_NDM-1_/*bla*_TEM_
	ICU	EC-84	*bla*_NDM-1_/*bla*_OXA-48_/*bla*_SHV_/*bla*_CTX-M_
		EC-32	*bla*_NDM-1_/*bla*_OXA-48_/*bla*_SHV_
		EC-35	*bla*_NDM-1_/*bla*_SHV_/*bla*_CTX-M_/*bla*_TEM_
		EC-24	*bla*_OXA-48_/*bla*_SHV_/*bla*_CTX-M_/*bla*_TEM_
	Pediatrics medicine	EC-42	*bla*_NDM-1_/*bla*_SHV_/*bla*_CTX-M_/*bla*_TEM_
		EC-20	*bla*_OXA-48_/*bla*_VIM_/*bla*_SHV_
Blood	Surgery	EC-76	*bla*_NDM-1_/*bla*_OXA-48_/*bla*_TEM_
		EC-93	*bla*_NDM-1_/*bla*_CTX-M_/*bla*_TEM_
		EC-19	*bla*_OXA-48_/*bla*_VIM_/*bla*_SHV_
		EC-28	*bla*_OXA-48_/*bla*_SHV_/*bla*_CTX-M_
	Nephrology	EC-25	*bla*_OXA-48_/*bla*_SHV_/*bla*_CTX-M_/*bla*_TEM_
		EC-36	*bla*_NDM-1_/*bla*_SHV_
	Pediatrics medicine	EC-18	*bla*_OXA-48_/*bla*_CTX-M_
	Oncology	EC-90	*bla*_NDM-1_/*bla*_OXA-48_/*bla*_SHV_
Tip-cells	Surgery	EC-05	*bla*_NDM-1_/*bla*_SHV_/*bla*_CTX-M_
		EC-03	*bla*_OXA-48_/*bla*_SHV_/*bla*_CTX-M_
		EC-96	*bla*_NDM-1_/*bla*_SHV_
	Medicine	EC-47	*bla*_NDM-1_/*bla*_SHV_
		EC-53	*bla*_OXA-48_/*bla*_SHV_
	Nephrology	EC-64	*bla*_OXA-48_/*bla*_SHV_/*bla*_CTX-M_
		EC-72	*bla*_NDM-7_/*bla*_SHV_
	ICU	EC-65	*bla*_NDM-1_/*bla*_SHV_/*bla*_CTX-M_
		EC-61	*bla*_OXA-48_/*bla*_SHV_
Pleural-fluid	Surgery	EC-54	*bla*_NDM-1_/*bla*_OXA-48_/*bla*_SHV_/*bla*_CTX-M_
		EC-41, EC-55	*bla*_NDM-1_/*bla*_SHV_
	Medicine	EC-85	*bla*_NDM-1_/*bla*_OXA-48_/*bla*_CTX-M_/*bla*_TEM_
		EC-59	*bla*_OXA-48_/*bla*_CTX-M_
	Chest medicine	EC-70	*bla*_NDM-1_/*bla*_OXA-48_/*bla*_SHV_/*bla*_CTX-M_
		EC-100	*bla*_NDM-1_/*bla*_SHV_
	Urology	EC-66	*bla*_OXA-48_/*bla*_SHV_
Tissue	Oncology	EC-62	*bla*_NDM-1_/*bla*_SHV_/*bla*_CTX-M_
		EC-52	*bla*_OXA-48_/*bla*_VIM_/*bla*_SHV_
		EC-87	*bla*_NDM-1_/*bla*_SHV_
Sputum	Chest medicine	EC-46, EC-74	*bla*_OXA-48_/*bla*_SHV_/*bla*_CTX-M_

**Table 2:** Antimicrobial resistance and genetic profile of NDM-positive *E. coli* isolates.

Clinical Strains	Resistance Profile	MLST	Replicon Type
EC-02, EC-14, EC-17, EC-23, EC-30, EC-33, EC-34, EC-35, EC-42, EC-45, EC-47, EC-56, EC-62, EC-75, EC-83	CPT, CFZ, CAZ, CTX, FOX, ATM, AMP, AMC, AK, TZP, TGC	ST405	IncFII, IncA/C, IncN, IncL/M
EC-05, EC-21, EC-22, EC-36, EC-38, EC-41, EC-44, EC-78, EC-86, EC-88, EC-98, EC-103	CPT, CFZ, CAZ, CTX, FOX, ATM, AMP, AMC, CIP, DO, SXT, TZP	ST405	IncFII, IncA/C, IncN, IncL/M
EC-49, EC-55, EC-71, EC-76, EC-90	CPT, CFZ, CAZ, CTX, FOX, ATM, AMP, AMC, AK, PB, CIP, TGC	ST405	IncFII, IncA/C, IncN, IncL/M
EC-07, EC-13, EC-15, EC-40, EC-68, EC-72, EC-77, EC-84, EC-87, EC-89, EC-95, EC-101,	CPT, CFZ, CAZ, CTX, FOX, ATM, AMP, AMC, DO, CIP, SXT, TZP	ST131	IncFII, IncA/C, IncL/M
EC-16, EC-27, EC-50, EC-67, EC-73, EC-85, EC-96, EC-97, EC-102	CPT, CFZ, CAZ, CTX, FOX, ATM, AMP, AMC, AK, TZP, SXT, TGC	ST131	IncFII, IncA/C, IncL/M
EC-09, EC-10, EC-12, EC-29, EC-31, EC-80, EC-104	CPT, CFZ, CAZ, CTX, FOX, ATM, AMP, AMC, PB, TGC, SXT, DO	ST10	IncFII, IncN
EC-32, EC-39, EC-65, EC-100	CPT, CFZ, CAZ, CTX, FOX, ATM, AMP, AMC, AK, TZP	ST101	IncFII, IncN
EC-43, EC-54, EC-70	CPT, CFZ, CAZ, CTX, FOX, ATM, AMP, AMC, TZP, CIP, DO, TGC	ST101	IncFII, IncN
EC-11, EC-57, EC-63, EC-93, EC-94	CPT, CFZ, CAZ, CTX, FOX, ATM, AMP, AMC, SXT, CIP	ST648	IncFII, IncN, IncFIIK

**Table 3:** Distribution of sequence types with resistance gene pattern.

MLST	Resistance Gene Pattern	No. of Isolates
ST405	*bla*_NDM-7_/*bla*_OXA-48_/*bla*_SHV_/*bla*_CTX-M_/*bla*_TEM_	EC-17
	*bla*_NDM-7_/*bla*_OXA-48_/*bla*_SHV_/*bla*_CTX-M_	EC-98
	*bla*_NDM-7_/*bla*_CTX-M_	EC-86
	*bla*_NDM-1_/*bla*_OXA-48_/*bla*_CTX-M_/*bla*_TEM_	EC-23, EC-56
	*bla*_NDM-1_/*bla*_OXA-48_/*bla*_SHV_/*bla*_CTX-M_	EC-75, EC-98
	*bla*_NDM-1_/*bla*_OXA-48_/*bla*_TEM_	EC-76
	*bla*_NDM-1_/*bla*_OXA-48_/*bla*_SHV_	EC-02, EC-33, EC-90
	*bla*_NDM-1_/*bla*_VIM_/*bla*_SHV_	EC-71, EC-103
	*bla*_NDM-1_/*bla*_SHV_/*bla*_CTX-M_/*bla*_TEM_	EC-35, EC-42
	*bla*_NDM-1_/*bla*_SHV_/*bla*_CTX-M_	EC-05, EC-34, EC-38, EC-44, EC-62
	*bla*_NDM-1_/*bla*_CTX-M_/*bla*_TEM_	EC-83, EC-88
	*bla*_NDM-1_/*bla*_CTX-M_	EC-14, EC-21, EC-22, EC-49
	*bla*_NDM-1_/*bla*_SHV_	EC-30, EC-36, EC-41, EC-45, EC-47, EC-55, EC-78
ST131	*bla*_NDM-7_/*bla*_OXA-48_/*bla*_SHV_/*bla*_CTX-M_	EC-89
	*bla*_NDM-7_/*bla*_SHV_/*bla*_CTX-M_	EC-50, EC-102
	*bla*_NDM-7_/*bla*_SHV_	EC-72
	*bla*_NDM-1_/*bla*_OXA-48_/*bla*_SHV_/*bla*_CTX-M_	EC-73, EC-84, EC-95
	*bla*_NDM-1_/*bla*_OXA-48_/*bla*_CTX-M_/*bla*_TEM_	EC-85, EC-97, EC-101
	*bla*_NDM-1_/*bla*_SHV_/*bla*_CTX-M_	EC-13, EC-15, EC-40, EC-67, EC-68, EC-77
	*bla*_NDM-1_/*bla*_SHV_	EC-27, EC-87, EC-96
	*bla*_NDM-1_/*bla*_CTX-M_	EC-16
	*bla*_NDM-1_/*bla*_TEM_	EC-07
ST10	*bla*_NDM-1_/*bla*_SHV_/*bla*_CTX-M_/*bla*_TEM_	EC-10, EC-29
	*bla*_NDM-1_/*bla*_SHV_/*bla*_CTX-M_	EC-09, EC-31, EC-104
	*bla*_NDM-1_/*bla*_CTX-M_/*bla*_TEM_	EC-12
	*bla*_NDM-1_/*bla*_CTX-M_	EC-80
ST101	*bla*_NDM-1_/*bla*_OXA-48_/*bla*_SHV_/*bla*_CTX-M_	EC-39, EC-54, EC-70
	*bla*_NDM-1_/*bla*_OXA-48_/*bla*_SHV_	EC-32
	*bla*_NDM-1_/*bla*_SHV_/*bla*_CTX-M_	EC-65
	*bla*_NDM-1_/*bla*_SHV_	EC-43, EC-100
ST648	*bla*_NDM-1_/*bla*_OXA-48_/*bla*_SHV_/*bla*_CTX-M_	EC-57
	*bla*_NDM-1_/*bla*_SHV_/*bla*_CTX-M_	EC-11, EC-94
	*bla*_NDM-1_/*bla*_CTX-M_/*bla*_TEM_	EC-93
	*bla*_NDM-1_/*bla*_CTX-M_	EC-63

**Table 4:** AgNPs and MEM interaction determined by ΣFIC values.

Sr. #	Strain ID	ΣFIC	Interpretation
1	EC-98 (*bla*_NDM-1_/*bla*_OXA-48_)	0.75	Synergism
2	EC-17 (*bla*_NDM-7_/*bla*_OXA-48_)	1	Synergism
3	EC-89 (*bla*_NDM-7_/*bla*_OXA-48_)	1	Synergism
4	EC-98 (*bla*_NDM-7_/*bla*_OXA-48_)	0.5	Synergism
5	EC-23 (*bla*_NDM-1_/*bla*_OXA-48_)	1	Synergism
6	EC-56 (*bla*_NDM-1_/*bla*_OXA-48_)	1.5	Indifferent
7	EC-73 (*bla*_NDM-1_/*bla*_OXA-48_)	1	Synergism
8	EC-75 (*bla*_NDM-1_/*bla*_OXA-48_)	1	Synergism
9	EC-50 (*bla*_NDM-7_)	0.75	Synergism
10	EC-86 (*bla*_NDM-7_)	0.5	Synergism
11	EC-12 (*bla*_NDM-1_)	0.75	Synergism
12	EC-32 (*bla*_NDM-1_)	1	Synergism
13	EC-42 (*bla*_NDM-1_)	1.2	Indifferent
14	EC-44 (*bla*_NDM-1_)	1	Synergism
15	EC-62 (*bla*_NDM-1_)	0.5	Synergism
16	EC-63 (*bla*_NDM-1_)	0.75	Synergism
17	EC-261	0.5	Synergism
18	EC-423	0.5	Synergism
19	EC-438	0.5	Synergism
20	EC-503	1	Synergism
21	EC-510	3	Antagonism
22	EC-587	2	Indifferent

synergism ≤ 1, indifferent 1.1 to 2.0, antagonism effect ≥ 2.

## Data Availability

The data used to support the findings of this study are available from the corresponding authors upon request.

## Supplementary Materials

The following supporting information can be downloaded at: https://www.mdpi.com/article/10.3390/microorganisms10112283/s1, Table S1: Nucleotide sequences for PCR and sequencing [12,13,14,15]; Table S2: Concentration of MEM and AgNPs
