# Supplementary material for: Characterization of Genomic Diversity among Carbapenem-Resistant Escherichia coli Clinical Isolates and Antibacterial Efficacy of Silver Nanoparticles from Pakistan
Source: Microorganisms. 2022 Nov 17;10(11):2283. doi: 10.3390/microorganisms10112283 (PMC9699514; doi:10.3390/microorganisms10112283)
Supplement: Supplementary file 1 [file microorganisms-10-02283-s001.zip › microorganisms-2001494-supplementary.pdf]

Table S1: Nucleotide sequences for PCR and sequencing

	Primer Sequences	Tm (^{o}C) / Amplicon (bp)
**Carbapenemases** [12, 13]		
bla$_{\text{KPC-2}}$	F: GCTACACCTAGCTCCACCTTC	55 / 989
	R: ACAGTGGTTGGTAATCCATGC	
bla$_{\text{NDM-1}}$	F: GGGCAGTCGCTTCCAACGGT	53 / 476
	R: GTAGTGCTCAGTGTCGGCAT	
bla$_{\text{NDM-1}}$	F: ATGGAATTGCCCAATATTATGC	50 / 813
	R: TCAGCGCAGCTTGTCGGCCAT	
bla$_{\text{VIM}}$	F: GATGGTGTTTGGTCGCATA	52 / 390
	R: CGAATGCGCAGCACCAG	
bla$_{\text{OXA-48}}$	F: GCGTGGTTAAGGATGAACAC	52 / 438
	R: CATCAAGTTCAACCCAACCG	
bla$_{\text{IMP}}$	F: GGAATAGAGTGGCTTAAYTCTC	52 / 232
	R: GGTTTAAYAAAACAACCACC	
**ESBLs** [14]		
bla$_{\text{SHV}}$	F: CTTTATCGGCCCTCACTCAA	55 / 237
	R: AGGTGCTCATCATGGGAAAG	
bla$_{\text{TEM}}$	F: CGCCGCATACACTATTCTCAGAATGA	55 / 445
	R: ACGCTCACCGGCTCCAGATTTAT	
bla$_{\text{CTX-M}}$	F: ATGTGCAGYACCAGTAARGTKATGGC	55 / 593
	R: TGGGTRAARTARGTSACCAGAAYCAGCGG	
**MLST**		
**PCR amplification (Tm 55°C)**[15]		
dinB	F: GTTTTCCCAGTCACGACGTTGTATGAGAGGTGAGCAATGCGTA	
	R: TTGTGAGCGGATAACAATTTCCGTAGCCCCATCGCTTCCAG	
icd2	F: GTTTTCCCAGTCACGACGTTGTAATTCGCTTCCCGGAACATTG	
	R: TTGTGAGCGGATAACAATTTCATGATCGCGTCACCAAAYTC	
pabB	F: GTTTTCCCAGTCACGACGTTGTAAATCCAATATGACCCGCGAG	
	R: TTGTGAGCGGATAACAATTTCGGTTCCAGTTCGTCGATAAT	
polB	F: GTTTTCCCAGTCACGACGTTGTAGGCGGCTATGTGATGGATTC	
	R: TTGTGAGCGGATAACAATTTCGGTTGGCATCAGAAAACGGC	
putP	F: GTTTTCCCAGTCACGACGTTGTACTGTTTAACCCGTGGATTGC	
	R: TTGTGAGCGGATAACAATTTCGCATCGGCCTCGGCAAAGCG	
trpA	F: GTTTTCCCAGTCACGACGTTGTAGCTACGAATCTCTGTTTGCC	
	R: TTGTGAGCGGATAACAATTTCGCTTTCATCGGTTGTACAAA	
trpB	F: GTTTTCCCAGTCACGACGTTGTACACTATATGCTGGGCACCGC	
	R: TTGTGAGCGGATAACAATTTCCCTCGTGCTTTCAAAATATC	
uidA	F: GTTTTCCCAGTCACGACGTTGTACATTACGGCAAAGTGTGGGTCAAT	
	R: TTGTGAGCGGATAACAATTTCCCATCAGCACGTTATCGAATCCTT	
**Sequencing**		
oF	GTTTTCCCAGTCACGACGTTGTA	
oR	TTGTGAGCGGATAACAATTTC	

Abbreviations: *bla*$_{\text{KPC}}$, *klebsiella pneumoniae* carbapenemase gene; *bla*$_{\text{NDM}}$, New Delhi metallo beta lactamase; beta *bla*$_{\text{OXA-48}}$, beta-lactamase oxacillinase 48 gene; *bla*$_{\text{VIM}}$, metallo-beta-lactamase verona integron gene; *bla*$_{\text{IMP}}$, beta-lactamase imipenemase gene; *bla*$_{\text{CTX-M}}$, beta-lactamase cefotaxime munich gene; *bla*$_{\text{SHV}}$, beta-lactamase sulfhydryl reagent variable gene; *bla*$_{\text{TEM}}$, beta-lactamase temoneira gene; *dinB,* DNA polymerase; *icdA*, isocitrate dehydrogenase; *pabB*, p-aminobenzoate synthase; *polB*, polymerase PolIII; *putP*, proline permease; *trpA*; tryptophan synthase subunit A; *trpB*, tryptophan synthase subunit B; *uidA*, beta-glucuronidase

Table S2: Concentration of MEM and AgNPs

MEM (µg/mL)	AgNPs (µg/mL)	MEM / AgNPs (µg/mL)
1024	10	512 / 10
512	5	256 / 5
256	2.5	128 / 2.5
128	1.25	64 / 1.25
64	0.625	32 / 0.625
32	0.312	16 / 0.312
16		
8		
